# Phase 1 dose-escalation trial combining sulfasalazine and stereotactic radiosurgery in patients with recurrent glioblastoma

**DOI:** 10.1016/j.redox.2026.104241

**Published:** 2026-05-30

**Authors:** Bente Sandvei Skeie, Alexander Richard Craig-Craven, Eli Renate Grüner, Martin Biermann, Frank Riemer, Sidsel Bragstad, Shahin Sarowar, Maziar Behbahani, Linda Sleire, Thomas Schwarzlmüller, Dorota Goplen, Jan Ingeman Heggdal, Jonathan P.S. Knisely, Michael Schulder, Geir Egil Eide, Christopher G. Filippi, Per Øyvind Enger

**Affiliations:** a Department of Neurosurgery, Haukeland University Hospital (HUS), Jonas Lies vei 65, Bergen, 5021, Norway; b Department of Biomedicine, University of Bergen (UiB), Jonas Lies vei 91, Bergen, Norway; c Department of Biological and Medical Psychology, UiB, Jonas Lies vei 91, Bergen, Norway; d Department of Clinical Engineering, HUS, Jonas Lies vei 65, Bergen, 5021, Norway; e Department of Physics and Technology, UiB, Jonas Lies vei 65, Bergen, 5021, Norway; f Mohn Medical Imaging and Visualization Center, Department of Radiology, HUS, Jonas Lies vei 65, Bergen, 5021, Norway; g Nuclear Medicine and PET Centre, Department of Radiology, HUS, Jonas Lies vei 65, Bergen, 5021, Norway; h Section for Radiology, Clinical Institute I, UiB, Jonas Lies vei 65, Bergen, 5021, Norway; i Department of Neurosurgery, Stavanger University Hospital, Armauer Hansens vei 30, Stavanger, 4011, Norway; j Laboratory Medicine and Pathology, HUS, Jonas Lies vei 65, Bergen, 5021, Norway; k Department of Oncology and Medical Physics, HUS, Jonas Lies vei 65, Bergen, 5021, Norway; l Department of Radiation Oncology, Amsterdam University Medical Center, Meibergdreef 9, Amsterdam, 1105 AZ, the Netherlands; m Department of Neurosurgery, Donald and Barbara Zucker School of Medicine at Hofstra/Northwell, 125 Community Dr, Great Neck, NY, 11021, USA; n Department of Radiology, The Hospital for Sick Children, 555 University Ave, Toronto, M5G 1X8, Canada

**Keywords:** Sulfasalazine, High grade glioma, Glioblastoma, Recurrence, Stereotactic gamma knife radiosurgery, Radiosensitizer

## Abstract

Glioblastoma radioresistance is linked to glutathione, a key antioxidant protecting against oxidative stress. Sulfasalazine inhibits the xCT antiporter mediating cystine uptake required for glutathione synthesis in glioma, potentiating radiotherapy in preclinical studies. This trial evaluated safety and therapeutic potential of combining sulfasalazine with stereotactic radiosurgery (SRS) for recurrent glioblastoma. Adults with recurrences were enrolled in a 3 + 3 dose-escalation trial receiving 1.5, 3.0, 4.5, or 6.0 g sulfasalazine orally for 3 days before SRS. Primary endpoint was safety. Secondary endpoints included changes in quality of life (QOL), tumor glutathione-levels, metabolism, and volumes assessed via Functional-Assessment-of-Cancer-Therapy-Brain-questionnaire, glutathione-edited-MR-spectroscopy,^11^C-Methionine-PET, and MRI. Eleven eligible patients treated with GKRS only due to closed research facilities served as concurrent controls for time to local tumor progression, progression-free survival (PFS), and overall survival (OS). Twelve patients were enrolled and followed until death or consent withdrawal (n = 1). Of 20 adverse events, two were grade 3 (transient lymphocytopenia), four grade 2, and fourteen grade 1. QOL remained stable for 6 months (p = 0.292). Tumor glutathione-levels and metabolic activity were reduced on day 3 (p = 0.010) and 1 month (p = 0.052), respectively. Best RANO-responses were objective (5/11), stable (5/11) or progressive (1/11) for participants vs. stable (2/11) or progressive (9/11) for controls, p = 0.001. Participants had longer local tumor control (median difference: 5.3 months, 95% CI: 1.0-9.6, p < 0.001) and PFS (1.7 months, 95% CI: 0.3-3.0, p = 0.009), with equivalent OS (p = 0.915). In conclusion sulfasalazine and SRS were well tolerated, reducing intratumoral glutathione-levels and associated with more durable local control than SRS alone supporting phase II investigation.

**Trial registration:**

NCT04205357.

## Highlights

• Glioblastoma recurrence is highly radioresistant and among the deadliest cancers.
• First trial testing sulfasalazine as radiosensitizer with stereotactic radiosurgery.
• Treatment was well tolerated with no severe or late toxic side effects.
• Short-course sulfasalazine suppresses glutathione levels to increase radiosensitivity.
• Intervention showed high objective response rate warranting further investigation.

## Graphical abstract

**Figure:**
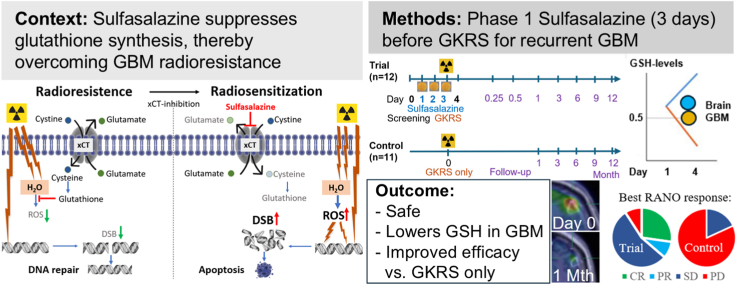

## 1 Introduction

Glioblastoma remains one of the most lethal brain tumors, with a median overall survival (OS) of only 14.6 months and virtually no long-term survivors [1]. Despite its intrinsic radioresistance, radiotherapy remains the most effective component of the standard-of-care regimen [2]. However, progression-free survival (PFS) is limited to 6.7 months [1]. Attempts to improve outcomes through radiation dose-escalation [3,4] or reirradiation for recurrent glioblastoma [5,6] have not improved survival. Thus, novel strategies to enhance the efficacy of radiotherapy [7] for glioblastoma are urgently needed.

Experimental data suggest that sulfasalazine may act as a radiosensitizer by inhibiting the cystine-glutathione (xCT) cell membrane antiporter, which is upregulated in several tumor types including glioblastoma [8,9]. xCT-inhibition blocks cystine uptake [10,11], a rate-limiting step in glutathione synthesis. Glutathione is a key intracellular antioxidant that protects tumor cells against reactive oxygen species induced by radiation [12].

Although sulfasalazine has not been clinically evaluated as a radiosensitizer, it has been tested for its antitumor potential in a retrospective phase I trial for newly diagnosed glioblastoma in combination with radiation therapy and temozolomide [13] and in a phase I/II trial as monotherapy for recurrent glioblastoma [14]. In both these trials long-term administration was associated with high rates of severe toxicity, including bone marrow suppression [13,14], and cerebral edema leading to early termination [14].

In this phase-I trial (NCT04205357), we investigated the safety and preliminary efficacy of a short, three-day sulfasalazine pretreatment regimen designed to deplete tumors of glutathione prior to single-session SRS in patients with recurrent glioblastoma.

## 2 Material and methods

### 2.1 Study design

This phase 1, open-label, non-randomized single-center trial followed a 3 + 3 dose-escalation design to determine dose limiting toxicity (DLT) and recommended dose of sulfasalazine. Dose escalation levels were 1.5 g, 3.0 g, 4.5 g, and 6.0 g of sulfasalazine for 3 consecutive days prior to Gamma Knife radiosurgery (GKRS). The sulfasalazine dose levels and three-day pretreatment schedule were selected based on available pharmacokinetic data [15,16] and prior clinical experience from a study by Robe et al., in which identical dose levels were administered to patients with recurrent glioblastoma [14] as detailed in the trial protocol [15]. Each dose level included a cohort of 3-6 participants [17]. The decision to escalate, de-escalate, or terminate the trial was made by an independent data safety monitoring committee. Each participant was monitored for adverse event (AE) for a minimum of one week before inclusion of the next patient.

### 2.2 Study participants

All patients with recurrent glioblastoma referred to our center were discussed at weekly multidisciplinary tumor board meetings including neurosurgeons, neuro-oncologists, neurologists and neuroradiologists. Retreatment strategies were determined on a case-by-case basis and included re-operation, stereotactic radiosurgery, conventional fractionated reirradiation, or systemic therapies alone or in combination. Factors favoring stereotactic radiosurgery included lesion diameter <3 cm, eloquent or deep-seated tumor location, and for patients for which surgical resection was associated with increased risk due to patient age, comorbidity, or reduced performance status.

The main trial inclusion criteria were adult patients with first or second relapse following Stupp-regimen for newly diagnosed glioblastoma, IDH wildtype with a recurrence amenable to GKRS less than 3 cm in diameter, a Karnofsky Performance Status (KPS) score ≥70, and adequate liver, renal, and hematological status. A complete list of inclusion and exclusion criteria are presented in the Supplementary Material. All participants provided written informed consent. One participant withdrew consent at 1 month follow-up, but safety data collected until consent withdrawal were used. None of the participants were lost to follow-up during the study.

### 2.3 Screening

During screening on Day -30-0 (D0) medical history including tumor histology at diagnosis (WHO, MGMT methylation and IDH mutation status) was collected. To assess tumor eligibility, ^11^C methionine (MET)-PET-MRI was undertaken to differentiate between recurrent tumor and radiation-induced changes from previous radiotherapy. Baseline KPS, health-related quality of life (QOL)-Functional Assessment of Cancer Therapy-Brain (FACT-Br) questionnaire [18] score, general/neurological status and laboratory screening to assure normal hematologic, hepatic, and renal function were obtained.

### 2.4 Intervention

A single daily dose of oral sulfasalazine was administered on Days 1-3 (D1-D3) prior to single session GKRS on Day 3 (D3) with Gamma Knife ICON (ELEKTA, Sweden). A prescription dose of 12 Gy to the contrast-enhancing tumor margin as defined on same-day MRI scan was used according to our department's standard dose-planning practices for recurrent glioblastoma [15,19]. The GammaPlan software (Elekta) was used for dose planning.

### 2.5 Assessments

#### 2.5.1 Adverse events

Participants were monitored for AEs for 30 days. Bloodwork, urine analysis, general/neurological examinations, and KPS-scoring were performed daily during trial treatment and in the outpatient clinic at one, two, and four weeks. Acute radiation induced AE were assessed on MRI prior to discharge (D4). AEs were graded according to the Common Terminology Criteria for Adverse Events (CTCAE)v5 criteria [20] and evaluated for causal relationship to sulfasalazine in combination with GKRS.

#### 2.5.2 Quality of life

KPS and QOL were assessed at D0, D4, one month (M1), three months (M3), then three-monthly via telephone interviews and FACT-Br questionnaire.

#### 2.5.3 Radiology

**Fig. 1:**
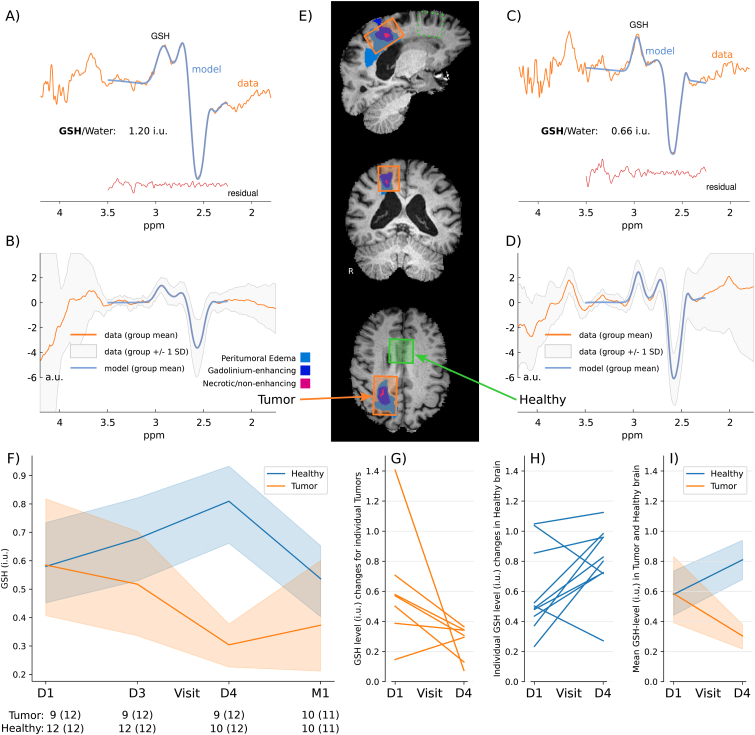
Glutathione-edited MR-spectroscopy data acquired in tumor (A, B) and healthy brain (C, D), showing a single spectrum and corresponding model fit from a representative participant in the upper panels (A, C), with group mean data, standard deviation (SD) and group mean model fit (all timepoints) presented in the lower panels (B, D). Data were acquired from voxels of nominally 27 ml, with the tumor voxel positioned to maximize overlap with the individual's tumor and the healthy voxel positioned in the midline anterior cingulate cortex, illustrated for a single representative patient in (E). Mean GSH-levels in tumor and healthy brain at D1 (prior to first SAS dose), D3 (after the third/last dose of SAS prior to GKRS), D4 (the day after GKRS) and M1 for 12 trial participants (F). Changes in GSH-levels for individual tumors (G) versus brain (H). GSH, Glutathione; SAS, Sulfasalazine; GKRS, Gamma Knife Radiosurgery; D, Day; M1, 1 month follow-up.

MR-Spectroscopy was acquired on D1, D3, D4, and at M1 to quantify glutathione levels using the MEGA-PRESS pulse sequence, see Supp. material, Table S1 and Fig. 1A–E. ^11^C-MET PET-MRI was performed at baseline (D1) to confirm tumor recurrence, as this modality has been shown to differentiate recurrent glioblastoma from pseudoprogression with a high positive predictive value of 93.9 % [21] and at the 1 M follow-up to assess changes in tumor and brain metabolism. MET-PET images were evaluated by a nuclear medicine physician according to the PET RANO 1.0 criteria [22]. Measurable PET-positive disease was defined as a maximum standardized uptake value (SUV_max_) ≥ 1.6 [23]. Additional PET-scans were conducted when needed to distinguish tumor recurrence from pseudoprogression.

**Fig. 2:**
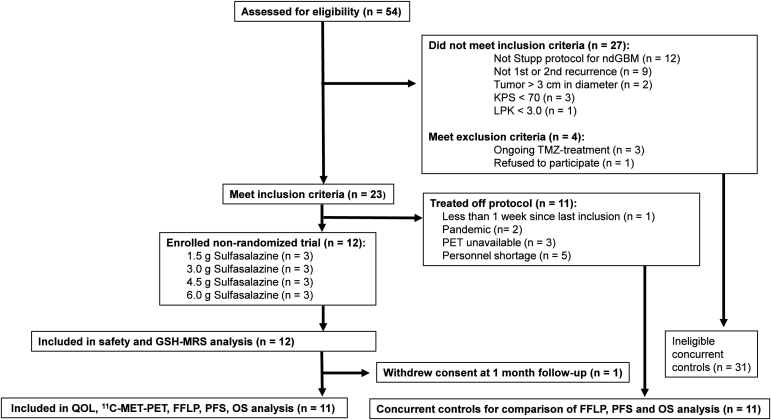
Flowchart of inclusion and exclusion of trial participants and concurrent control groups. ndGBM = newly diagnosed Glioblastoma; KPS: Karnofsky Performance Status; LPK = leucocyte count; TMZ = Temozolomide; 11C-MET-PET = 11C-Methionine positron emission tomography; GSH-MRS = Glutathione Magnetic resonance Spectroscopy.

Standard MRI prior to admission and during follow-up at M1, M3 and then three-monthly were co-registered with stereotactic MRI in GammaPlan and evaluated by a neuroradiologist. Progressive disease (PD) according to modified RANO-criteria [[24], [25], [26]] was noted as preliminary PD and confirmed PD if sustained on MRI follow-up. Conversely, if the lesion later remained stable (SD), or displayed minimal (MR), partial (PR), or complete response (CR), it was registered as pseudoprogression. Best tumor response, freedom from local progression (FFLP; time to progression of the treated lesions or last MRI without local failure), distant progression, and progression-free survival (PFS; time to death or local/distant tumor progression whichever occurred first) were registered.

#### 2.5.4 Treatment at progression

Upon local or distant tumor progression, participants were evaluated at multidisciplinary meetings for additional treatments.

#### 2.5.5 Non-randomized controls

As this was a single-arm phase I study, a randomized control group was not included. Although retrospective series have reported outcomes following SRS for glioblastoma, available data are heterogeneous with limited relevance for IDH-wildtype recurrences treated with single-session GKRS after completion of the Stupp protocol. Therefore, a contemporary non-randomized control cohort was established from patients with glioblastoma recurrences who fulfilled all clinical eligibility criteria for trial screening, based on referral information, telephone prescreening, medical history, and review of available imaging and histopathology (including IDH and MGMT status), and who were treated with GKRS using identical dose-planning practices. However, these patients did not proceed to formal trial screening and enrollment including protocol-mandated laboratory testing and PET imaging.

Specifically, trial inclusion was paused during protocol-mandated safety observation periods during dose escalation (≥1 week following enrollment of the most recently treated patient), and during PET-center downtime due to technical repairs, COVID-19-related restrictions, and institutional research closures (mid-June to mid-August and the final two weeks of December). Consequently, baseline study-specific procedures were not performed in these patients. The selection process is summarized in the study flowchart (Fig. 2).

### 2.6 Statistical analysis

Descriptive statistics were used to characterize baseline patient, tumor, and treatment characteristics. Medians or means were estimated for continuous variables, and proportions (%) were estimated for categorical data. The primary endpoint was safety. DLT was defined as the dose where >1/3 experienced AE grade ≥3 [27]. FFLP, PFS and OS were calculated using the Kaplan-Meier method and as difference between groups with a confidence interval (CI) on absolute difference. Changes over multiple time points in tumor volume, KPS, FACT-Br, SUV_max_ and glutathione levels were evaluated using the mixed linear model with Šidák adjustment for multiple comparisons. For glutathione analysis, interaction between location (tumor vs. normal brain), timepoint (relative to baseline) and sulfasalazine dose were calculated. The trial and the control groups were compared using the Wilcoxon-Mann-Whitney test, the exact chi-square test also including test for trend, and the log-rank-test. Comparisons were also performed after propensity score adjustments [28] based on clinically relevant baseline variables: age, KPS, MGMT methylation status, time from diagnosis to recurrence, and baseline tumor volume. Given the limited sample size and loss of matched observations when models included more than four covariates, a reduced model including age, KPS, baseline volume and time from diagnosis to recurrence was used in the final matched analyses. SPSS version 29.0 and R were used for statistical analysis.

## 3 Results

### 3.1 Participants

From March 1, 2020, to August 30, 2022, 54 patients were referred to and accepted for GKRS for recurrent glioblastoma, of which 12 were enrolled in the trial (Fig. 2). One participant withdrew consent prior to the 1-month follow-up MRI, but data collected until consent withdrawal were used. All participants had confirmed WHO grade 4 glioblastomas (IDH wild type) at diagnosis and received treatment according to the Stupp-protocol. However, only eight participants (66.7%) completed all six scheduled temozolomide maintenance doses. Most recurrences (n = 8) were located at the margin of the surgical cavity, two in the corpus callosum, and two distant from the primary tumor and outside of the previous radiation-field.

#### 3.1.1 Non-randomized controls

A total of 54 patients with recurrent glioblastoma were treated with GKRS during the trial period. Of the 42 patients treated outside protocol, 11 were eligible but not included in the trial due to reasons described in the method section and served as controls. Control patients were younger than trial participants (mean age 51.1 years vs. 61.5 years, p = 0.009), all other baseline characteristics were similar (Table 1). Individual baseline characteristics are presented in Supp. Table S2.

**Table 1:** Baseline characteristics for 12 trial participants and 11 concurrent controls.

Variable	Group									
	Trial			Control			P-values			
	(n = 12)			(n = 11)			WMW	T-test	SW-T	SW-C
Age (years), *mean, median, range*	61.5	61.0	[54, 69]	51.1	51.0	[35, 66]	0.007	0.009	0.245	0.623
Sex (males), *n (%)*	7	(58)		4	(36)		0.414∗		n.a.	n.a.
TMZ cycles, *mean, median, range*	5.2	6.0	[2, 6]	5.2	6·0	[3, 6]	0.990	0.978	<0.001	<0.001
MGMT promoter methylation, *n (%)*	5	(42)		7	(64)		0.414∗		n.a.	n.a.
Months from surgery, *mean median, range*	17.8	11.5	[5, 46]	12.8	13·0	[7, 30]	0.705	0.309	0.083	0.021
1st recurrence, *n (%)*	9	(75)		9	(82)		1.00∗			n.a.
Infield of prior RT field, *n (%)*	10	(83)		10	(91)		1.00∗			n.a.
No tumors, *mean, median, range*	1.25	1.00	[1, 4]	1.64	1.00	[1, 4]	0.220	0.343	<0.001	<0.001
Tumor volume (cm^3^), *mean, median, range*	2.31	1.62	[0.09, 6.76]	2.83	2.41	[0.29, 8.46]	0.487	0.606	0.049	0.115
Asymptomatic recurrence, *n (%)*	9	(75)		11	(100)		0.217∗			n.a.
Steroidal medication (mg), *mean, median, range*	0.38	0.00	[0, 0.5]	0.05	0.00	[0, 0.5]	0.863	0.346	<0.001	<0.001
KPS, *mean, median, range*	79·2	80.0	[70, 90]	83.6	90.0	[70, 90]	0.193	0.196	0.018	0.002
GKRS dose (Gy), *mean, median, range*	12.0	12·0	[12, 12]	12.4	12.0	[8, 18]	0.568	0.640	n.a.	0.078
SAS dose, *mean, median, range*	3.75	4.75	[1.5, 6.0]	0.00	0.00	[0, 0]	<0.001	<0.001	0.078	n.a.
QOL FACT-BR, *mean, median, range*	52	51	[41, 66]	Not measured			n.a.	n.a.	0.105	n.a.
Intense PET-signal, n (%)	10	(83)		Not measured			n.a.	n.a.	n.a.	n.a.

*Abbreviations:* TMZ = temozolomide; ndGBM = newly diagnosed glioblastoma; MGMT = methylguanine-DNA methyltransferase; RT = radiotherapy; KPS = Karnofsky's Performance Status (scale 0-100); QOL FACT-BR = Quality of Life Functional Assessment of Cancer Therapy for Brain cancer patients scale (0-100); PET = Positron emission tomography; SAS = sulfasalazine; M = male; F = female; GKRS = Gamma Knife Radiosurgery; Md = median; P-value = two-tailed for comparing trial patients with controls; WMW = Exact Wilcoxon-Mann-Whitney test; T-test = Gosset's unpaired *t*-test with separate variances; SW-T = Shapiro Wilk's test for normality in the trial group; SW-C = Shapiro-Wilk's test for normality in the control group; n.a. = not applicable; ∗ = Two-sided exact Pearson’ s chi-square test.

### 3.2 Safety

Sulfasalazine was administered successfully under the supervision of the study nurse with no DLT observed. A total of 20 AEs were recorded, of which two (10%) were grade 3, four (20%) grade 2 and 14 (70%) grade 1 (Table 2). The number of AEs per participant was not correlated with sulfasalazine dose (p = 0.245). However, AE severity increased with higher sulfasalazine doses (p = 0.043). One participant in each of the two highest dose cohorts, experienced asymptomatic grade 3 lymphocytopenia on D3, a known, common side effect of sulfasalazine per the Summary of Product Characteristics. Both cases normalized within 24 h without intervention. All grade 2 AEs occurred in the highest dose cohort. One participant experienced three grade 2 AEs; temporary headache, hypersomnia, and memory impairment, which were managed with dexamethasone. MRI showed no evidence of tumor swelling or edema. One experienced short-term pain following removal of the stereotactic frame, managed with paracetamol. The most frequently reported grade 1 AE was mild gastrointestinal symptoms.

**Table 2:** Adverse events for 12 trial participants.

ID	SAS dose	CTCAE term	Symptom	Start date	Duration	Grade^a)^	Intervention	SmPC SAS
T1	1.5 g	1) Surgical or medical procedure	Bleeding from one pin site after frame removal	D3	<1h	1	Compression	Uncommon
T2	1.5 g	None						
T3	1.5 g	1) Gastrointestinal system disorders	Diarrhea	D2	<1h	1	None	Very common
		2) Surgical or medical procedure	Bleeding from pin site after frame removal	D3	<1h	1	Compression	Uncommon
T4	3.0 g	1) Nervous system disorder	Taste disorder	D3	24 h	1	None	Common
T5	3.0 g	1) Gastrointestinal system disorder:	Gastric distress/Bloating	D15	5 days	1	None	Common
T6	3.0 g	1) Eye system disorder	Blurred vision	D4	3 days	1	None	Uncommon
		2) Nervous system disorder	Taste disorder	D4	6 days	1	None	Common
		3) Infections and infestations	Upper respiratory infection	W2	6 days	1	None	Uncommon
T7	4.5 g	None						
T8	4.5 g	1) Gastrointestinal system disorder	Nausea	D3	<1 h	1	None	Very common
		2) Gastrointestinal system disorder	Vomiting	D3	<1h	1	None	Common
T9	4.5 g	1) Surgical or medical procedure	Bleeding from one pin site after frame removal	D3	<1 h	1	Compression	Uncommon
		2) Investigations	Lymphocyte count decreased <0.5-0.2 x 10e9/L	D3	<24 h	3	None	Common
T10	6.0 g	1) Investigations	Lymphocyte count decreased <0.5-0.2 x 10e9/L	D3	<24 h	3	None	Common
T11	6.0 g	1) Gastrointestinal system disorders	Dyspepsia	D3	<1 h	1	None	Common
		2) Gastrointestinal system disorders	Nausea	D4	5 days	1	None	Common
		3) Nervous system disorder	Headache	D5	14 days	2	Dexamethasone	Uncommon
		4) Nervous system disorder	Hypersomnia	M1	5 days	2	Dexamethasone	Uncommon
		5) Nervous system disorder	Memory impairment	M1	5 days	2	Dexamethasone	Uncommon
T12	6.0 g	1) Surgical or medical procedure	Headache after frame removal	D3	<1 h	2	Paracetamol	Uncommon
		2) Ear and Labyrinth disorder	Tinnitus	D4	<1 h	1	None	Common
Total						20		

*Abbreviations:* CTCAE = Common Terminology Criteria for Adverse Events; SAS = sulfasalazine; SmPC = Summary of Product Characteristics.

a) p-value = 0.245 for independence of number of AEs of SAS dose levels (Chi-square test for linear-by-linear trend). P-value = 0.043 for comparison of highest rank AE between the different dose levels (Chi-square test for linear-by-linear trend).

### 3.3 Quality of life

**Fig. 3:**
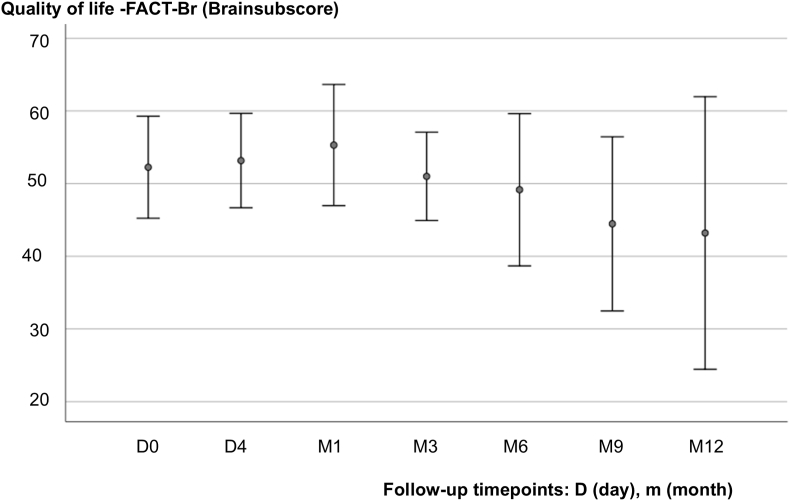
Changes in Functional Assessment of Cancer Therapy - Brain (FACT-Br) Scores and KPS Scores for 12 months follow-up of the 12 trial participants.

FACT-Br scores remained stable during the treatment period, with a mean of 52.3 (SD 11.1) at baseline (D0) and 53.2 (SD 10.2) on D4 (Fig. 3 and Supp. Table S3). A modest improvement was observed at one month (mean 55.3, SD 11.7). QOL was maintained at three (mean 51.0, SD 8.5) and six months (mean 49.2, SD 11.3). However, participants reported a significant decline in QOL at nine (mean 44.5, SD 13.0) and 12 months (mean 43.2, SD 15.1). KPS remained unchanged through the three-month follow-up but declined from the six-month follow-up onward.

### 3.4 Glutathione levels

At baseline, mean glutathione levels were identical in tumor tissue and normal brain (Fig. 1F–I, see Supp.Table S4). Tumor-glutathione-levels decreased significantly from a baseline mean of 0.59 international units (i.u.) to 0.31 i. u. on D4, representing a mean reduction of −0.281 i. u. (95% CI: −0.531 to −0.031; p = 0.027). In contrast, normal brain tissue exhibited a significant increase in glutathione levels over the same period, rising from 0.58 i. u. at baseline to 0.81 i. u. on D4, with a mean increase of 0.19 i. u. (95% CI: 0.035 to 0.350; p = 0.026). This divergence between tumor and normal brain glutathione levels was significant at all post-treatment time points (D3: p = 0.010; D4: p = 0.008; M1: p = 0.048) independent of sulfasalazine dose. Tumor-glutathione-levels remained suppressed at 1-month follow-up, whereas levels in normal brain tissue returned to baseline.

### 3.5 MET-PET-imaging

**Fig. 4:**
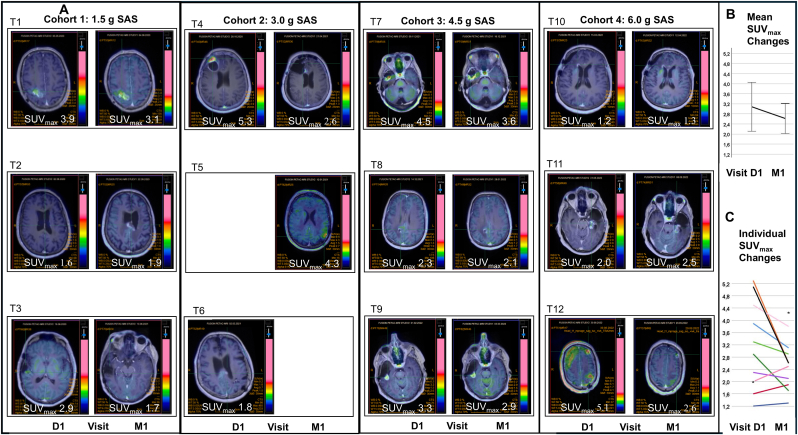
^11^C-Methionine PET images at baseline (D1) and follow-up (M1) for trial participants T1 to T12 (A). Mean changes in SUV_max_ from D1 to M1 (B) and changes in SUV_max_ for all individual trial participants (C). SAS, Sulfasalazine; PET, Positron emission tomography; SUV, standardized uptake volume; D, Day, M, month.

Baseline MET-PET-imaging was successfully acquired for 11 of 12 participants (92%, Fig. 4A). Of these, 10 (91%) exhibited measurable PET-positive disease at baseline. One lesion was PET-negative (SUV_max_: 1.2) but was interpreted as tumor recurrence by a neuroradiologist due to significant tumor growth from 0.4 cm^3^ to 0.7 cm^3^ since referral (Fig. 4A, T10).

Follow-up MET-PET scans were obtained in 11 trial participants (92%), with one participant withdrawing consent (Fig. 4A, T6). Reduced metabolic tumor activity was observed in 7 of 10 participants with both baseline and follow-up MET-PET-scans (Fig. 4A and B and C), with a mean change in SUV_max_ from baseline to M1 post treatment of −0.78 (95% CI: −1.57 to 0.01) showing a borderline significant reduction (p = 0.052). The best RANO-PET responses of the trial treated lesions were PET-CR in three, PET-PR in one and PET-SD in six. Within the PET-SD-group (<30% increase or decrease in SUV_max_), three trial treated lesions still demonstrated a reduction in SUV_max_ ranging from −9% to −21%, though not meeting the threshold for PET-PR.

### 3.6 Best RANO response and duration of response

**Fig. 5:**
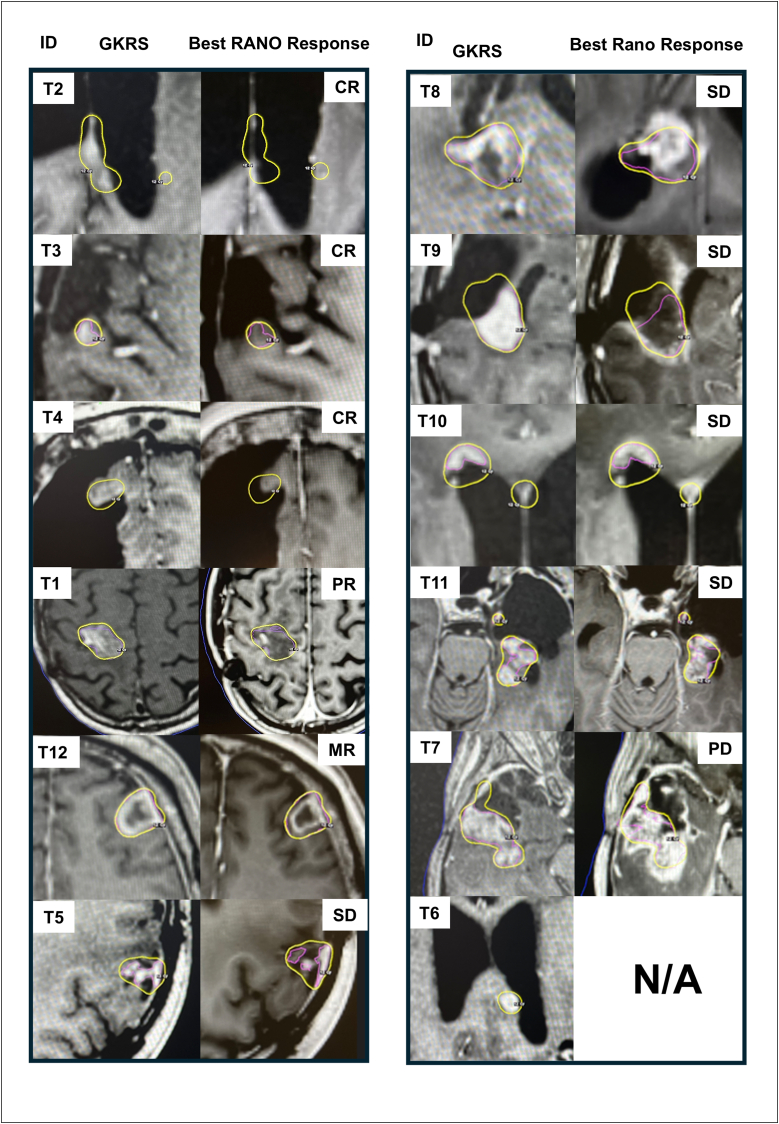
MRI at baseline and best RANO response during follow-up for 12 trial participants T1-T12. GKRS, Gamma Knife radiosurgery; T, trial; RANO, Response Assessment in Neuro-Oncology; CR, Complete response; PR, Partial response; MR, Minimal response; SD, Stable disease, PR, Progressive disease.

**Fig. 6:**
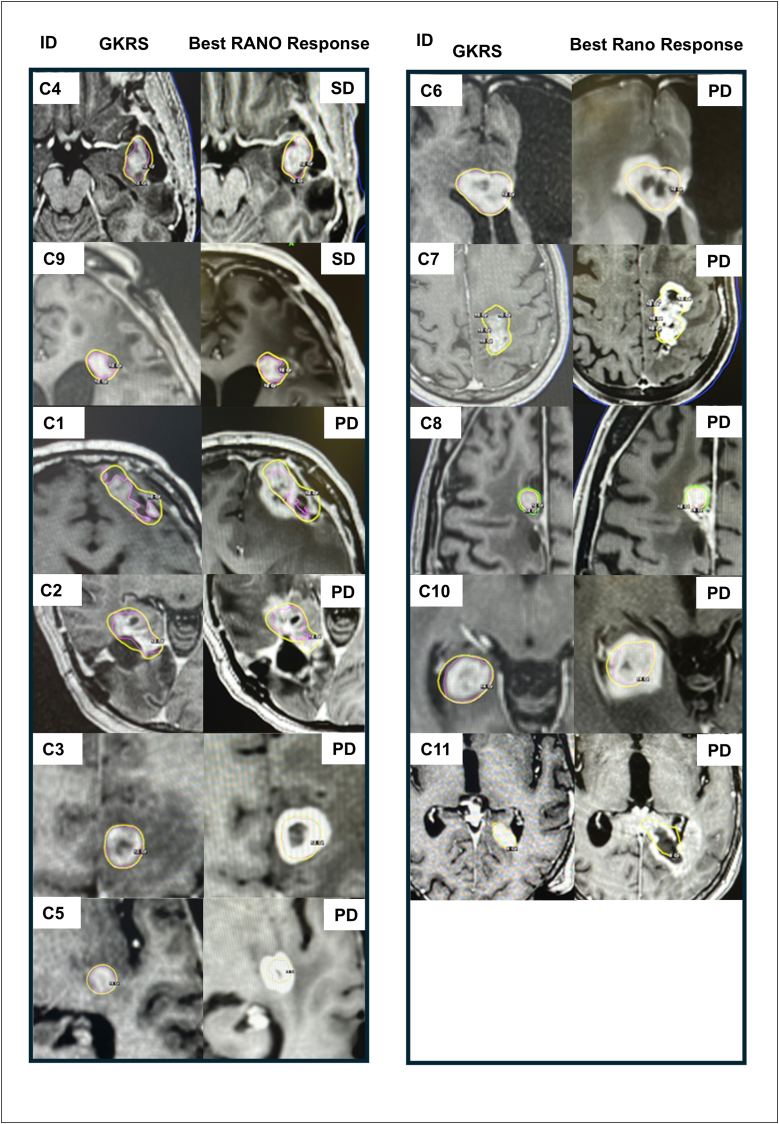
MRI at baseline and best RANO response during follow-up for 11 concurrent control patients C1–C11. GKRS, Gamma Knife radiosurgery; C, control; RANO, Response Assessment in Neuro-Oncology; CR, Complete response; PR, Partial response; MR, Minimal response; SD, Stable disease, PR, Progressive disease.

**Fig. 7:**
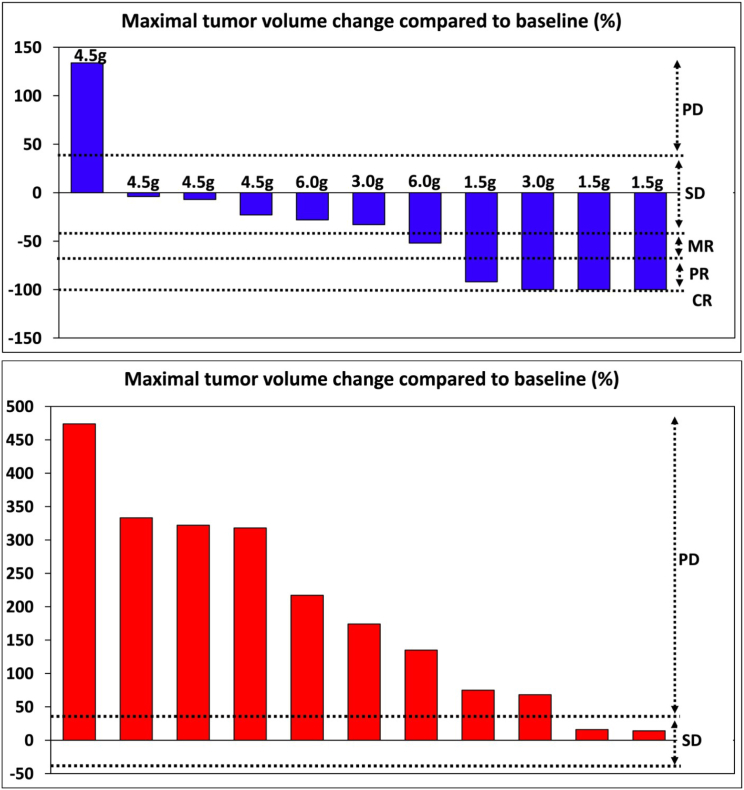
Waterfall plots for best RANO response in trial (upper panel) and control (lower) patients. For trial participants, sulfasalazine doses are annotated. RANO, Response Assessment in Neuro-Oncology; CR, Complete response; PR, Partial response; MR, Minimal response; SD, Stable disease, PR, Progressive disease.

An objective response was achieved for 5 of 11 trial participants (45.5%): CR in 3 (27.2%), PR in 1 (9.1%), and MR in 1 (9.1%, Fig. 5). SD was observed in 5 trial participants as best response (45.5%), all of whom still exhibited tumor shrinkage ranging from −28% to −7% compared to baseline. In contrast, none of the control patients had an objective response (Fig. 6), two displayed SD (18.2%) but still exhibited increased tumor volumes. PD was noted as best response in 1 trial participant (9.1%) versus 9 of 11 controls (81.8%; p = 0.001, Fig. 7). Ultimately, the GKRS-treated lesion progressed in 7 of 11 trial participants (63.6%) compared to all 11 controls (p = 0.027).

**Fig. 8:**
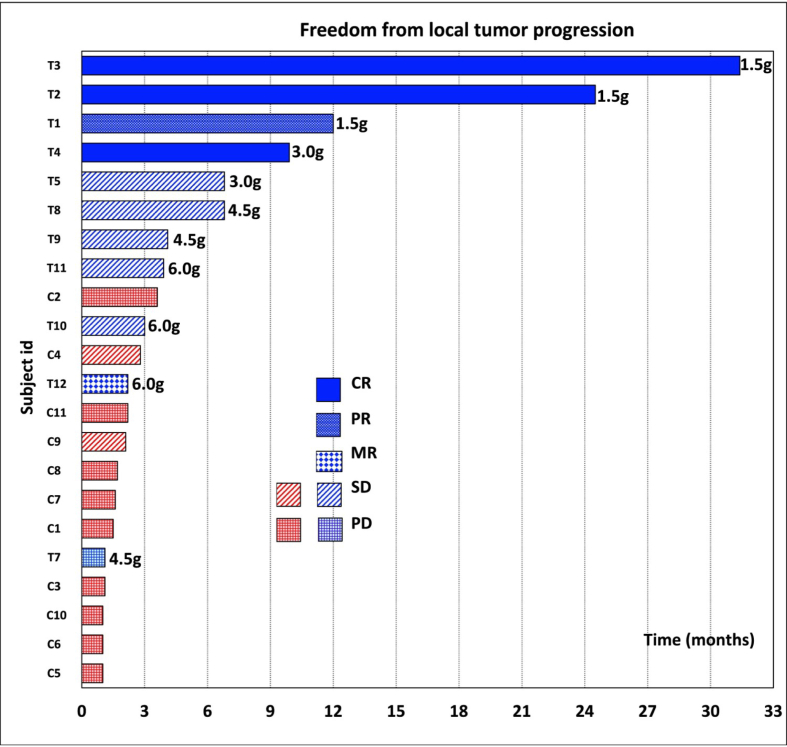
Swimmer's plot demonstrating duration of treatment response (freedom from local tumor progression), each bar representing trial participant (blue) and controls (red) are annotated according to subject id and sulfasalazine dose (trial participants). RANO, Response Assessment in Neuro-Oncology; CR, Complete response; PR, Partial response; MR, Minimal response; SD, Stable disease, PR, Progressive disease.

**Fig. 9:**
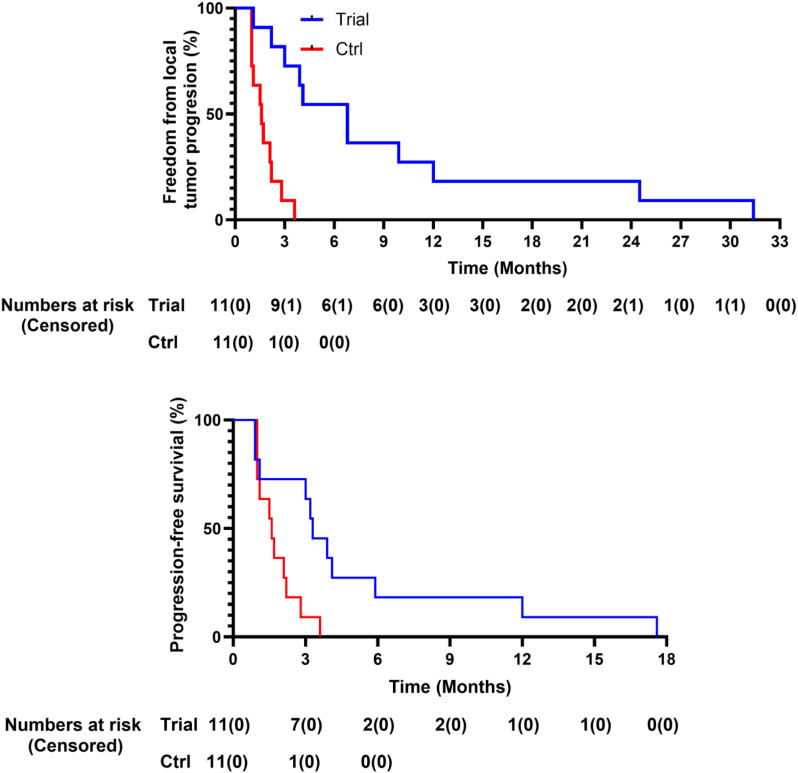
Kaplan-Meier curves illustrating freedom from local tumor progression (upper panel) and progression free survival times (lower panel). SAS, Sulfasalazine; T, trial; C, control; CR, Complete response; PR, Partial response; MR, Minimal response; SD, Stable disease, PR, Progressive disease.

Median FFLP was significantly longer in the trial group; 6.9 months versus 1.6 months in controls, with a difference of 5.3 months (95% CI: 1.0 to 9.6, p < 0·001), Fig. 8, Fig. 9 (upper panel). Trial participants with complete or partial response had the longest FFLPs, ranging from 9.9 to 31.4 months and none of the complete responding tumors re-occurred.

Distant recurrences occurred in 9 of 11 (81.8%) trial participants and 6 of 11 controls (54.5%, p = 0.183). Still, median PFS was longer in the trial group; 3.3 versus 1.6 months for controls with a difference of 1.7 months (95% CI: 0.3 to 3.0, p = 0.009, Table 3), Fig. 9 (lower panel).

**Table 3:** Comparison of treatment outcomes for 12 trial participants and 11 contemporary control patients.

Measure:	Best response (RANO criteria)	FFLP (months)	Distant failure	PFS (months)	Local failure	PsP	Time PsP	Steroid use Increased during follow-up	OS ndGBM (months)	OS rGBM (months)
Group	% PD	Median	%	Median	%	%	Median	% Yes	Median	Median
Control	81.8	1.6	45.5	1.6	100	9.1	>12	90.9	25.5	11.5
Trial	9.1	6.9	81.8	3.3	63.6	54.5	5	83.6	24.0	11.3
P-value	0.001^a)^	<0.001^b)^	0.183^c)^	0.009^b)^	0.027^c)^	0.063^c)^	0.037^b)^	0.214^d)^	0.424^b)^	0.915^b)^
P-value	**0.031^e)^**	**0.013^g)^**	**0.375^f)^**	**0.313 ^g)^**	**n.c.**	**0.375^f)^**	**0.165^g)^**	**n.c.**	**0.313^g)^**	**0.739^g)^**

*Abbreviations:* RANO = Response Assessment in Neuro-Oncology which has 4 levels; CR = complete response = 1; PR/MR = partial/minimal response = 2; SD = Stable disease = 3; PD = Progressive disease = 4; FFLP = freedom from local disease progression; LF = Local failure; PFS = Progression free survival; n.c. = could not be calculated; PsP = Pseudoprogression; OS = overall survival; ndGBM = newly diagnosed glioblastoma; rGBM = Recurrent GBM.

a) Chi-square test for linear trend in 2×4 table; b) Log-rank test; c) Exact chi-square test; d) Chi-square test for 2×3 table; e) *Exact Wilcoxon Signed-Rank test* after 1:1 propensity score matching (n = 9 pairs) based on age, KPS, time from diagnosis to recurrence, and baseline volume at GKRS; f) *Exact McNemar's test of symmetry* in a 2×2 table after 1:1 propensity score matching (n = 9 pairs) based on age, KPS, time from diagnosis to recurrence, and baseline volume at GKRS; g) Cox-regression *stratified* for rank after 1:1 propensity score matching (n = 9 strata) based on age, KPS, time from diagnosis to recurrence, and baseline volume at GKRS.

Only nine patients could be successfully matched using a model including age, KPS, baseline volume and time from diagnosis to recurrence (Table 3). In this matched cohort, the objective local response rate and FFLP remained significantly superior in trial participants compared with controls whereas PFS was not significantly different between groups.

No trial participants or controls required resection for radionecrosis. Pseudoprogression was observed more frequently in trial participants (6/11; 54.5%) than controls (1/11; 9.1%), although this difference did not reach statistical significance, p = 0.063. All pseudoprogressions occurred within the high-dose fractionated radiotherapy volume. Pseudoprogression was detected at median 3.0 months (95% CI: 1.45 to 4.55; range: 2–6 months) and resolved within a median of 2.5 months (range: 1–4 months). Individual imaging and clinical outcomes are presented in Supp. Table S5.

### 3.7 Survival

Median OS from the time of GKRS did not differ significantly between the trial and control groups; 11.3 versus 11.5 months, respectively, with a difference of −0.2 months (95% CI: −7.9 to 7.5, p = 0.915). The 6-month, 1-year and 2-year OS-rates were 91%, 46% and 18% for the trial group, and 73%, 36% and 9% for the control group. OS from primary diagnosis, was 24.0 months (95% CI: 16.4 to 31.6) for trial participants and 25.5 months (95% CI: 13.6 to 37.5) for controls (p = 0.424). Outcomes for glioblastoma patients treated with GKRS for recurrence during the trial period but ineligible for trial prescreening and/or inclusion (Fig. 2) were also inferior to those of enrolled participants. Ineligible patients had an FFLP of 3.0 months (95% CI 2.6–3.7; p < 0.001) and a PFS of 2.6 months (95% CI 1.4–3.7; p = 0.025), while median OS was 8.4 months (95% CI 5.1–11.7; p = 0.965).

## 4 Discussion

Short-term sulfasalazine administration as radiosensitizer in conjunction with SRS was safe. To minimize systemic exposure, we employed a brief 3-day sulfasalazine regimen aimed at depleting tumors of glutathione prior to single-session GKRS. AEs were predominantly mild and transient, with only two cases of grade 3 lymphocytopenia observed at higher sulfasalazine doses. Gastrointestinal grade 1 AEs were common and consistent with sulfasalazine's known toxicity profile. Notably, toxicity increased with higher drug doses. Whereas sulfasalazine serum concentrations were not assessed, these analyses are included in the protocol for our planned double-blind placebo-controlled multi-center phase-2 trial investigating the efficacy of 1.5 g sulfasalazine in combination with GKRS (54 participants in each arm). However, it is important to note that radiation-induced side effects often manifest beyond the typical 30-day AE reporting window used in phase 1 trials [29]. This limitation may underestimate late toxicities such as radiation necrosis which remains a significant concern in re-irradiation strategies. Notably, over 90% of glioblastoma recurrences occur within regions previously exposed to high-dose fractionated radiotherapy. The reported rates of radiation necrosis following SRS vary widely [19,[30], [31], [32]] depending on the dosimetry applied. In a retrospective study using 18 Gy prescription dose, 40 % of the patients experienced AE due to radiation [30]. In contrast, employment of a conservative dose of 12 Gy in the present trial did not lead to any cases of radiation necrosis either in trial participants or contemporary controls. Importantly, patient-reported quality of life was sustained for trial participants throughout the first six months of treatment, indicating a low risk of late-onset radiation toxicity. These findings support the feasibility and tolerability of a 3-day sulfasalazine pretreatment followed by GKRS as a radiosensitization strategy.

Tumor volume reduction was observed in 10 of 11 trial participants, with five meeting RANO criteria for objective response. In contrast, all control tumors treated off protocol increased in size. Notably, objective responses were associated with substantially longer response duration than stable disease. The median local response duration of 6.9 months compared favorably with our contemporary control groups. Moreover, it was 2 and 2.8 months longer, respectively than the local response durations reported in two previous retrospective studies despite longer imaging control intervals and inclusion of patients with grade III glioma and newly diagnosed glioblastoma in the retrospective studies [33,34]. Interestingly, although not statistically significant, trial participants had exhibited a higher incidence of pseudoprogression within the first 6 months compared to concurrent controls suggesting a more pronounced local tissue reaction [31] in trial treated tumors. Furthermore, MET-PET demonstrated reduced metabolic activity in the majority of lesions. Notably, PET-CR was registered for all three tumors with complete response on anatomical MRI. Thus, the superior RANO responses in trial participants when compared to controls, with a high rate of objective response and pseudoprogression, was supported by our PET data. Although our findings suggest that adding sulfasalazine to GKRS may enhance efficacy consistent with preclinical data [8], the observed radiologic signs of a potential radiosensitizing effect should be interpreted cautiously given the non-randomized phase I design, which was not powered for efficacy assessment.

Although all participants who achieved durable local tumor control ultimately developed new distant recurrences, PFS was significantly prolonged in the trial cohort in the unadjusted analysis, suggesting a potential overall therapeutic benefit. After propensity score matching, the objective response and FFLP remained significantly better in the trial group. Whereas PFS was not significantly longer, these matched analyses should be interpreted cautiously give the small sample size with only nine patients matched for a limited number of covariates [35,36]. Bevacizumab has been shown to prolong PFS but not OS in glioblastoma patients [37]. Notably, none of the trial participants received bevacizumab before or within the first two months following GKRS, during which 9 of 11 control patients experienced disease progression. In the control group PFS was reached at 1.6 months (Supp. Table S5). The difference in PFS between trial group and controls can therefore not be ascribed to bevacizumab treatment since no trial participants had received bevacizumab at this point. Despite a favorable local response duration, the median OS times were similar for trial participants and contemporary controls. This may be due to the small sample size, lack of randomization, and age difference with the control cohort being, on average, 10 years younger. Notably, age is a well-known prognostic factor for glioblastoma with higher age consistently associated with shorter survival [38].

MR-Spectroscopy revealed a reduction in intratumoral glutathione levels, even for the lowest sulfasalazine dose. Although sulfasalazine has limited penetration across an intact blood-brain barrier (BBB), a defining feature of glioblastoma is disruption of the BBB [39] due to tumor-induced neo-angiogenesis characterized by abnormal pericyte coverage [40], loss of astrocytic end feet [41], degradation of the basal membrane [42], emergence of endothelial cell fenestrations [43], dysregulation and loss of tight junctions [44], collectively leading to increased permeability [40]. This feature is routinely exploited in clinical practice through gadolinium-enhanced MRI, where contrast agents that do not cross an intact BBB accumulate in glioblastoma tissue [45]. A pilot clinical trial in patients with glioma-associated epilepsy demonstrated that 1 g oral sulfasalazine administration was associated with reduced peritumoral glutamate levels on magnetic resonance spectroscopy, with greater reductions observed in tumors with higher xCT expression [46] supporting CNS target engagement by sulfasalazine. Since glutathione normally protects tumors from oxidative stress by reducing DNA damage, and promoting survival, these findings implicate glutathione depletion as a mechanism for radiosensitization. We observed increased glutathione levels in normal brain which may be surprising given its intact BBB. However, this may reflect a compensatory mechanism mediated through tumor-brain crosstalk rather than a direct effect of sulfasalazine in brain parenchyma. Glutathione levels in gliomas are typically lower than in normal brain tissue [47,48]. Although MR-Spectroscopy is limited by voxel size, improved analytical methods to edit MRS multiplex spectra with overlapping resonance signals have made it possible to filter out and identify single metabolites that can be quantified with high precision [49] including glutathione levels in the human brain for several disease entities, including gliomas [47], Parkinson's disease [50] and epilepsy [51]. The concentrations measured in this study fall within the validated detection range of the MEGA-PRESS sequence [[52], [53], [54], [55]].

Radiation remains the most critical and effective component in the multimodal management of glioblastoma [56], yet it is often accompanied by devastating side effects. Our approach capitalizes not only on the intratumoral xCT-antiporter upregulation, but on single session treatment and the precision of GKRS to deliver a higher biologically effective dose selectively to tumor tissue when compared to SRS alone. In the current trial, repeat SRS for new lesions was performed with GKRS alone although repeat short-course sulfasalazine combined with repeat radiosurgery is feasible and may further enhance disease control. This approach has been incorporated into the protocol for the planned randomized controlled phase II trial. However, bone marrow suppression associated with long-term treatment of sulfasalazine limits its use as a radiosensitizer during fractionated radiotherapy concomitant with temozolomide for newly diagnosed glioblastoma. Thus, structure-optimized sulfasalazine derivatives with reduced toxicity and preserved efficacy have been developed, with several candidates demonstrating preclinical activity [57]. Apart from allowing for longer treatment duration in conjunction with fractionated radiotherapy and temozolomide, they may also enable dose de-escalation strategies to protect surrounding brain tissue without compromising therapeutic outcomes. Moreover, the xCT-antiporter is overexpressed in other radioresistant tumors, suggesting this regimen can also be applied to other cancers [9] and extracranial SRS.

Combining sulfasalazine with other compounds that increase oxidative stress via cancer cell-specific mechanisms may further enhance the anti-tumor efficacy of radiotherapy. Preclinical studies have shown that pharmacological ascorbate potentiates radiotherapy by increasing intracellular H_2_O_2_ and O_2_**^.^**^-^ production and disrupting redox-active iron metabolism in glioblastoma cell lines, without affecting non-malignant cells [58,59]. Of note, ascorbate has also been evaluated in clinical phase I and II trials in patients with non-small cell lung cancer and newly diagnosed glioblastoma receiving radiochemotherapy with little toxicity and favorable outcome compared to historical controls [58,60]. Moreover, MRI-based imaging supports a mechanistic link between ascorbate treatment and changes in redox-active iron and could serve as a tool to identify patients who are more likely to respond to ascorbate [61], and to monitor treatment response [62]. Although sulfasalazine and ascorbate affect tumor redox biology through different mechanisms, reducing ROS neutralization and enhancing ROS production respectively, their effects are likely synergistic and provide a rationale for combining these drugs to potentiate radiotherapy.

## 5 Summary

In conclusion, the trial regimen was well tolerated. Despite the limited sample size, mechanistic imaging demonstrated successful intratumoral glutathione depletion and reduced metabolic activity at a sulfasalazine dose of 1.5 g. Moreover, clinical response data suggest that this dose is sufficient to radiosensitize glioblastoma cells and achieve more durable local tumor control compared with stereotactic radiosurgery alone, with a low risk of adverse effects. These findings warrant confirmation in a randomized phase II trial using 1.5 g sulfasalazine as the recommended dose [63]. Given sulfasalazine's off-patent status and low cost, successful validation could enable rapid clinical implementation.

## Ethical approval statement

The trial was approved by the regional ethical committee (REK vest 6834) and the European Medicines Agency (EUDRACT No.2019-002110-40). The privacy rights of human subjects have been observed and informed consent was obtained from all trial participants. All treatments and follow-up data were recorded using electronic Case Report Forms (Viedoc), monitored and evaluated by the DMC. The trial was planned in collaboration with the national brain tumor organization, and the scientific integrity was overseen by a steering committee.

## Data availability statement

The data generated in this study is not publicly available due to information that could compromise patient consent, but deidentified data are available upon reasonable request from the corresponding author.

## Funding

This trial was supported by the 10.13039/100008730Norwegian Cancer Society (Grant reference number: 2019/6834).

## CRediT authorship contribution statement

**Bente Sandvei Skeie:** Conceptualization, Data curation, Funding acquisition, Investigation, Methodology, Project administration, Supervision, Visualization, Writing – original draft, Writing – review & editing. **Alexander Richard Craig-Craven:** Conceptualization, Data curation, Formal analysis, Investigation, Methodology, Visualization, Writing – review & editing. **Eli Renate Grüner:** Conceptualization, Data curation, Formal analysis, Investigation, Methodology, Supervision, Visualization, Writing – review & editing. **Martin Biermann:** Data curation, Formal analysis, Investigation, Methodology, Visualization, Writing – review & editing. **Frank Riemer:** Data curation, Formal analysis, Investigation, Methodology, Visualization, Writing – review & editing. **Sidsel Bragstad:** Conceptualization, Data curation, Investigation, Methodology, Writing – review & editing. **Shahin Sarowar:** Data curation, Investigation, Writing – review & editing. **Maziar Behbahani:** Investigation, Writing – review & editing. **Linda Sleire:** Conceptualization, Methodology, Writing – review & editing. **Thomas Schwarzlmüller:** Investigation, Writing – review & editing. **Dorota Goplen:** Investigation, Writing – review & editing. **Jan Ingeman Heggdal:** Investigation, Writing – review & editing. **Jonathan P.S. Knisely:** Conceptualization, Writing – review & editing. **Michael Schulder:** Conceptualization, Writing – review & editing. **Geir Egil Eide:** Formal analysis, Investigation, Writing – original draft. **Christopher G. Filippi:** Investigation, Writing – review & editing. **Per Øyvind Enger:** Conceptualization, Investigation, Methodology, Supervision, Visualization, Writing – original draft, Writing – review & editing.

## Declaration of competing interest

None of the authors have received financial compensation related to their contributions to this work from pharmaceutical companies or other agencies. Jan Ingeman Heggdal serves as a consultant to Elekta. None of the authors have other disclosures or conflicts of interest related to this work.
