# Supplementary material for: Phase 1 dose-escalation trial combining sulfasalazine and stereotactic radiosurgery in patients with recurrent glioblastoma
Source: Redox Biol. 2026 May 30;95:104241. doi: 10.1016/j.redox.2026.104241 (PMC13251504; doi:10.1016/j.redox.2026.104241)
Supplement: Multimedia component 1 [file mmc1.docx]

**Supplementary Material**

Bente S Skeie, et al. **Phase 1 dose-escalation trial combining sulfasalazine and stereotactic radiosurgery in patients with recurrent glioblastoma**

**Supplementary Data and Results**

**Table of Contents Page Numbers**

[**Supplementary Methods** 2](#_Toc219371213)

[List of Inclusion Criteria: 2](#_Toc219371214)

[List of Exclusion Criteria: 3](#_Toc219371215)

[MR spectroscopy Glutathione (GSH)-edited data acquisition 3](#_Toc219371216)

[Supp. Table S1. MRSinMRS checklist of GSH-edited data acquisition: 4](#_Toc219371217)

[**Supplementary Results** 5](#_Toc219371218)

[Supp. Table S2. Baseline characteristics for trial participants and concurrent controls 5](#_Toc219371219)

[Supp. Table S3. Functional Assessment of Cancer Therapy - Brain (FACT-Br) and KPS Scores 6](#_Toc219371220)

[Supp. Table S4: Changes in Glutathione-levels in tumor versus brain on MR-spectroscopy 6](#_Toc219371221)

[Supp Table S5. Individual outcomes for trial participants and concurrent controls 7](#_Toc219371222)

[**Supplemetary References** 8](#_Toc219371223)

# **Supplementary Methods**

## List of Inclusion Criteria:

1. Histologically verified glioblastoma multiforme (GBM, World Health Organization [WHO] grade 4) with recurrence (first or second relapse, all subtypes) based on the Response Assessment in Neuro-Oncology (RANO) criteria.
2. Prior standard therapy for newly diagnosed GBM consisting of surgery, standard fractionated radiotherapy (fRT) to 60 Gray (Gy) concomitant with Temozolomide (TMZ).
3. Has been informed of other treatment options.
4. Must be eligible for Gamma Knife Radiosurgery (GKRS).
5. Tumor size ≤ 3 centimeters (cm) in diameter (≤ 15 cubic centimeters [cm³]) on magnetic resonance imaging (MRI) dated no more than 30 days before stereotactic radiosurgery (SRS) treatment.
6. Must be at least 18 years of age.
7. Must be ambulatory with a Karnofsky Performance Status (KPS) score ≥ 70.
8. Life expectancy > 12 weeks.
9. The laboratory parameters described in the material and methods must be within the reference areas, or if outside of the reference area, evaluated by the Principal Investigator (PI) to be safe to go through the study protocol.
10. Laboratory parameters for vital functions should be in the normal reference range. Laboratory abnormalities that are not clinically significant are generally permitted, except for the following laboratory parameters, which must be within the ranges specified:
    - Hematology: White Blood Cell count (WBC): ≥ 3.0 x 10⁹/L, Platelet count: ≥ 100 x 10⁹/L, Hemoglobin (Hb): ≥ 100 grams per liter (g/L)
    - Total bilirubin level: < 1.5 times the Upper Limit of Normal (ULN) (except in patients with Gilbert’s Syndrome who must have a total bilirubin less than 51.3 micromoles per liter [µmol/L])
    - Aspartate Aminotransferase (ASAT) or Alanine Aminotransferase (ALAT) < 3 times the ULN
    - Creatinine < 1.5 times the ULN
    - Normal prothrombin time / international normalized ratio (PT INR) < 1.4
    - Absolute Neutrophil Count (ANC): ≥ 1 x 10⁹/L without the support of filgrastim.
11. More than four weeks must have elapsed since any prior systemic therapy at the time the patient receives the preparative regimen, and patients’ toxicities must have recovered to a grade 1 or less. Patients may have undergone minor surgical procedures within the past 3 weeks, as long as all toxicities have recovered to grade 1 or less or as specified in the eligibility criteria.
12. Signed informed consent and expected cooperation of the patients for the treatment and follow-up must be obtained and documented according to International Council for Harmonization, Good Clinical Practice (ICH GCP), and national/local regulations.

## List of Exclusion Criteria:

1. Allergy to sulfa drugs.
2. Adverse reactions to salicylates.
3. Known hypersensitivity to sulfasalazine, its metabolites, or any of the excipients (Povidone; Maize starch; magnesium stearate; colloidal silicon dioxide).
4. Eligible for alternative standard treatments with temozolomide.
5. Treatment with sulfasalazine after glioblastoma diagnosis.
6. Participation in a pharmacokinetic trial within 4 weeks.
7. Participation in an immunotherapy trial within 4 weeks.
8. History of psychological symptoms affecting the ability to consent to and/or fulfill the protocol.
9. Other malignant diseases and multiple sclerosis.
10. Pregnant or breast-feeding patients.
11. Porphyria.
12. Kidney or liver deficiencies.
13. Glucose-6-Phosphate Dehydrogenase (G-6-PD) deficiency.
14. Severe allergy or bronchial asthma.
15. History of erythema multiforme.
16. Significant heart failure (New York Heart Association [NYHA] class III-IV) or renal failure (Chronic Kidney Disease [CKD] stage 3-5).
17. Intestinal or urinary obstruction.
18. Any reason why, in the opinion of the investigator, the patient should not participate (e.g., not able to comply with study procedures).

## MR spectroscopy Glutathione (GSH)-edited data acquisition

MR Spectroscopy data were acquired at multiple time points to evaluate the effects of sulfasazine treatment on GSH levels. Measurements were taken at baseline prior to trial drug administration (Day 1), after the last dose of sulfasazine but before GKRS (Day 3), 24 hours post-GKRS (Day 4), and at one month following trial treatment (Month 1). GSH-edited data were acquired using a MEGA-PRESS sequence(1), on a 3T MR/PET (Siemens Biograph mMR), from voxels located in the tumor (nominally 27 mL, with placement tailored to the individual tumor) and in healthy tissue (27 mL voxel in the midline anterior cingulate cortex, with placement tailored to the individual tumor and consistent between sessions Figure 1A-E). Data were processed using Gannet(2) 3.3.2, with spectral alignment disabled; GSH estimates scaled to water and reported in institutional units (i.u.), were taken for statistical analysis. Linear mixed effects models were used to assess longitudinal effects on GSH (across timepoints, within subject), and interactions between location (healthy/tumor), sulfasazine dose-level and time point. Sequence parameters including quality metrics and rejection criteria are detailed in the MRSinMRS checklist(3) in eTable1.

## Supp. Table S1. MRSinMRS checklist of GSH-edited data acquisition:

| Site (name or number) | Haukeland University Hospital |
| --- | --- |
| **1. Hardware** |  |
| a. Field strength [T] | 3 T |
| b. Manufacturer | Siemens |
| c. Model (software version if available) | Biograph mMR, VE11P |
| d. RF coils: nuclei (transmit/receive), number of channels, type, body part | mMR head/neck matrix coil (1H receive), 12/20 channels active |
| e. Additional hardware | N/A |
| **2. Acquisition** |  |
| a. Pulse sequence | MEGA-PRESS (GSH editing, Universal implementation(1) |
| b. Volume of interest (VOI) locations | Subject-specific intra-tumoral and contralateral healthy tissue |
| c. Nominal VOI size [cm^3^, mm^3^] | Nominally 27 mL (30 x 30 x 30 mm^3^), some individual adaption |
| d. Repetition time (T_R_), echo time (T_E_) [ms, s] | T_R_ = 2000 ms, T_E_ = 80 ms |
| e. Total number of excitations or acquisitions per spectrum  In time series for kinetic studies   1. Number of averaged spectra (NA) per time point 2. Averaging method (e.g. block-wise or moving average) 3. Total number of spectra (acquired/in time series) | 256 transients total, alternating edit-ON/-OFF |
| f. Additional sequence parameters (spectral width in Hz, number of spectral points, frequency offsets)  If STEAM:, mixing time (T_M_)  If MRSI: 2D or 3D, FOV in all directions, matrix size, acceleration factors, sampling method | Spectral width 4000Hz, 4096 data points  15 ms editing pulses at 4.56 ppm (edit-ON) and 7.5 ppm (edit-OFF) |
| g. Water suppression method | CHESS |
| h. Shimming method, reference peak, and thresholds for “acceptance of shim” chosen | 3D-DESS |
| i. Triggering or motion correction method (respiratory, peripheral, cardiac triggering, incl. device used and delays) | N/A |
| **3. Data analysis methods and outputs** |  |
| a. Analysis software | Gannet 3.3.2 |
| b. Processing steps deviating from quoted reference or product | Spectral alignment disabled to improve reliability in the presence of extremely strong lipid signal and potentially weak/absent major reference peaks |
| c. Output measure (e.g. absolute concentration, institutional units, ratio), processing steps deviating from quoted reference or product | Water-referenced estimates for GSH, *without* adjustment for voxel tissue content (traditional segmentation and assumed relaxation rates not suitable in tumor context) |
| d. Quantification references and assumptions, fitting model assumptions | N/A |
| **4. Data quality** |  |
| a. Reported variables (SNR, linewidth (with reference peaks)) | SNR GSH 8.05 [3.03,12.9] (healthy), 4.16 [2.25,8.83] (lesion)  SNR NAA 152 [99.7,247] (healthy), 77.4 [29.3,153] (lesion)  SNR Cr 130 [84.4,215] (healthy), 63.1 [20.9,114] (lesion)  FWHM GSH 10.1 [5.46,14.7] (healthy), 11.8 [5.15,23.11] (lesion)  FWHM NAA 11.6 [8.66,21.67] (healthy) 18.1 [11.4,26.7] (lesion)  FWHM Cr 10.2 [7.90,16.8] (healthy), 16.1 [10.2,22.8] (lesion)  Reported as median [95% confidence interval]; FWHM in Hz |
| b. Data exclusion criteria | FWHM GSH > 30 Hz; SNR GSH < 2 |
| c. Quality measures of postprocessing model fitting (e.g. CRLB, goodness of fit, SD of residual) | FitError GSH > 50%; any parameter estimate < 0  Extreme outliers (> 3 x median absolute deviation evaluated from baseline acquisitions (timepoint D1) on healthy side) |
| d. Sample spectrum | See Figure 1 |

# **Supplementary Results**

## Supp. Table S2. Baseline characteristics for trial participants and concurrent controls

| ID | Age  (y) | Sex | No of TMZ cycles | MGMT  methylation | Time surgery  (month) | 1^st^ or 2^nd^ recurrence  (treatment) | Infield of prior RT-field | No tumors | Tumor Volume  (cm^3^) | Symptomatic recurrence | Steroidal medication  (mg) | KPS | Gy | SAS dose  (g) | QOL FACT-Br a) | PET  SUVmax a) |
| --- | --- | --- | --- | --- | --- | --- | --- | --- | --- | --- | --- | --- | --- | --- | --- | --- |
| T1 | 68 | M | 6 | No | 10 | 1^st^ | Yes | 1 | 6.76 | Yes | 4 | 80 | 12 | 1.5 | 63 | 3.9 |
| T2 | 61 | M | 6 | Yes | 19 | 1^st^ | Yes | 1 | 0.46 | Yes | 0 | 70 | 12 | 1.5 | 50 | 1.6 |
| T3 | 54 | M | 6 | Yes | 46 | 1^st^ | Yes | 1 | 0.09 | No | 0 | 70 | 12 | 1.5 | 60 | 2.9 |
| T4 | 55 | F | 6 | Yes | 42 | 2^nd^  1^st^ (surgery) | Yes | 1 | 0.34 | No | 0 | 70 | 12 | 3.0 | 65 | 5.3 |
| T5 | 67 | M | 6 | Yes | 31 | 1^st^ | Yes | 1 | 1.17 | No | 0 | 80 | 12 | 3.0 | 66 | - |
| T6 | 67 | F | 6 | Yes | 20 | 1^st^ | No | 1 | 0.17 | No | 0 | 80 | 12 | 3.0 | 44 | 1.8 |
| T7 | 55 | F | 3 | No | 32 | 2^nd^  1^st^ (surgery) | Yes | 1 | 5.77 | Yes | 0 | 70 | 12 | 4.5 | 36 | 4.5 |
| T8 | 57 | F | 5 | No | 8 | 1^st^ | Yes | 1 | 2.08 | No | 0 | 80 | 12 | 4.5 | 52 | 2.3 |
| T9 | 69 | M | 6 | No | 11 | 1^st^ | Yes | 1 | 3.38 | No | 0 | 90 | 12 | 4.5 | 42 | 3.3 |
| T10 | 66 | F | 6 | No | 12 | 1^st^ | Yes | 4 | 0.70 | No | 0 | 90 | 12 | 6.0 | 65 | 1.2 |
| T11 | 61 | M | 2 | No | 5 | 1^st^ | Yes | 1 | 4.77 | No | 0 | 80 | 12 | 6.0 | 43 | 2.0 |
| T12 | 61 | M | 4 | No | 9 | 2^nd^  1^st^ (GKRS) | No | 1 | 2.07 | No | 0.5 | 90 | 12 | 6.0 | 41 | 5.1 |
| Mean | 61.5 | 64 % | 5·2 | 42 % | 17.8 | 75 % | 83 % | 1.25 | 2.31 | 75 % | 0.38 | 79.2 | 12 | 3.75 | 52 | 3.1 |
| Md(unit) | 61.0 | (M) | 6.0 | (Yes) | 11.5 | (1^st^) | infield | 1.00 | 1.62 | asymptomatic | 0.00 | 80.0 | 12 | 3.75 | 51 | (SD 1.4) |
| C1 | 50 | F | 3 | Yes | 10 | 1st | Yes | 1 | 2.54 | No | 0 | 80 | 12 | - | - | - |
| C2 | 51 | F | 4 | Yes | 7 | 1^st^ | Yes | 1 | 4.63 | No | 0 | 80 | 12 | - | - | - |
| C3 | 35 | F | 6 | No | 7 | 1^st^ | No | 1 | 0.86 | No | 0 | 70 | 12 | - | - | - |
| C4 | 49 | F | 5 | No | 8 | 2^nd^  1^st^ (surgery) | Yes | 1 | 1.49 | No | 0 | 90 | 12 | - | - | - |
| C5 | 53 | M | 6 | No | 13 | 1^st^ | Yes | 2 | 0.27 | No | 0 | 90 | 18 | - | - | - |
| C6 | 35 | F | 6 | Yes | 30 | 1^st^ | Yes | 1 | 3.24 | No | 0 | 90 | 12 | - | - | - |
| C7 | 57 | F | 3 | Yes | 13 | 1^st^ | Yes | 4 | 8.46 | No | 0 | 80 | 10 | - | - | - |
| C8 | 66 | M | 6 | Yes | 13 | 1^st^ | Yes | 2 | 1.65 | No | 0 | 80 | 14 | - | - | - |
| C9 | 44 | M | 6 | No | 18 | 1^st^ | Yes | 2 | 0.65 | No | 0 | 90 | 12 | - | - | - |
| C10 | 59 | M | 6 | No | 10 | 1^st^ | Yes | 1 | 2.41 | No | 0.5 | 70 | 14 | - | - | - |
| C11 | 63 | F | 6 | Yes | 14 | 2^nd^  1^st^ (GKRS) | Yes | 3 | 4.96 | No | 0 | 90 | 8 | - | - | - |
| Mean | 51.1 | 36 % | 5.2 | 55 % | 12.8 | 82 % | 91 % | 1.64 | 2.83 | 100 % | 0.05 | 83.6 | 12.4 | - | - | - |
| Md(unit) | 51.0 | (M) | 6.0 | (Yes) | 13.0 | (1^st^) | infield | 1.00 | 2.41 | asymptomatic | 0.00 | 90.0 | 12 | - | - | - |
| P-value | **0.009** | 0.414^*^ | 0.990 | 0.414^*^ | 0.705 | 1.000^*^ | 1.000^*^ | 0.220 | 0.487 | 0.217^*^ | 0.863 | 0.193 | 0.568 | n.a. | n.a. | n.a. |

*Abbreviations:* TMZ = Temozolomide; ndGBM = newly diagnosed glioblastoma multiforme; MGMT = methylguanine-DNA methyltransferase; RT = radiotherapy ; KPS = Karnofsky’s Performance Status (scale 0-100); QOL FACT-BR = Quality of Life Functional Assessment of Cancer Therapy for Brain cancer patients scale (0-100); PET = Positron emission tomography; SUVmax = standardized maximum uptake values; SAS = sulfasalazine; M = male; F = female; GKRS = Gamma Knife Radiosurgery; Md = median; P-value = two-sided test comparing trial patients with controls using the Wilcoxon-Mann-Whitney test and *Pearson’s exact chi-square test a) Not measured for control patients; n.a. = not applicabl

## Supp. Table S3. Functional Assessment of Cancer Therapy - Brain (FACT-Br) and KPS Scores

Results from mixed linear regression analyses of quality of life (FACT-BRCS) and KPS durimg 12 months follow-up of the 12 trial participants

| **Outcome**  **variable** | **Follow-up times** | | | | | | |
| --- | --- | --- | --- | --- | --- | --- | --- |
|  | **D0** | **D4** | **M1** | **M3** | **M6** | **M9** | **M12** |
| FACT-BRCS | n = 12 | n = 12 | n = 10 | n = 10 | n = 7 | n = 7 | n = 5 |
| Mean  (SD) | 52.3  (11.1) | 53.2  (10.2) | 55.3  (11.7) | 51.0  (8.5) | 49.2  (11.3) | 44.5  (13.0) | 43.2  (15.1) |
| p-value | Ref | 1.000 | 0.988 | 0.937 | 0.292 | **0.007** | **0.006** |
| KPS score | n = 12 | n = 12 | n = 10 | n = 10 | n = 8 | n = 8 | n = 6 |
| Mean  (SD) | 79·2  (7.9) | 79.2  (7.9) | 78.0  (9.2) | 79.0  (8.8) | 66.3  (7.4) | 62.5  (8.9) | 58.3  (11.7) |
| p-value | Ref | 1.000 | 1.000 | 1.000 | **0.018** | **0.001** | **< 0.001** |

*Abbreviations:* FACT-BRCS = Quality of Life Functional Assessment of Cancer Therapy for Brain cancer patients Scale (0-100); KPS = Karnofsky’s Performance Status (0-100); Mean = observed mean; SD = standard deviation; p-value = t-test of estimated regression coefficient in a mixed linear model Sidak adjusted for multiple comparisons, D = Day, M = Month

## Supp. Table S4: Changes in glutathione-levels in tumor versus brain on MR-spectroscopy

Results from mixed linear regression analyses of differences in glutathione levels in tumors versus healthy brain measured on D1, D3, D4 and M1 for 12 trial participants

| **Outcome**  **variable** | **Follow-up times** | | | |
| --- | --- | --- | --- | --- |
|  | **D1** | **D3** | **D4** | **M1** |
| Location |  |  |  |  |
| Tumor | n = 9 | n = 9 | n = 9 | n = 10 |
| Normal brain (reference) | n = 12 | n = 12 | n = 10 | n = 10 |
| Location*Time point |  |  |  |  |
| coefficient  (95%CI) | 0 | -0.546  (-0.961 to -0.132) | -0.579  (-1.009 to -0.150) | -0.504  (-1.005 to – 0.005) |
| p-value | Reference | 0.010 | 0.008 | 0.048 |

*Abbreviations:* D = Day; M = month; Location*Time point = interaction between location and time point for Glutathion levels in tumor versus brain; CI = confidence interval, D = Day, M = Month.

## Supp Table S5. Individual outcomes for trial participants and concurrent controls

| ID | Best response (RANO) | FFLP (months) | LF  (Yes/No) | Distant failure  (Yes/No) | PFS (months) | PsP  (Yes/No) | Time PsP  (months) | Steroids use  Increased(Yes/No) | Bevacizumab (months after GKRS to bevacizumab treatment) | OS ndGBM  (month) | OS from GKRS  rGBM  (month) | QOL FACT-Br  (D0 -last f-up) | Best PET RANO Response of trial lesion | PET SUVmax (baseline – best response) |
| --- | --- | --- | --- | --- | --- | --- | --- | --- | --- | --- | --- | --- | --- | --- |
| T1 | PR | 12.0 | Yes | No | 12.0 | Yes | 3-6 | stable | Yes (6) | 25.2 | 14.43 | 63 - 61 | PET-SD | 3.9 - 3.1 |
| T2 | CR | 24.5 | No | Yes | 17.6 | Yes | 3-6 | No | No | 47.3 | 27.10 | 50 - 37 | PET-CR | 1.6 - 1.9 |
| T3 | CR | 31.4 | No | Yes | 3.2 | Yes | 3-6 | No | Yes (21) | 81.5 | 34.53 | 60 - 55 | PET-CR | 2.9 - 1.7 |
| T4 | CR | 9.9 | No | Yes | 0.9 | No | - | Yes | No | 54.2 | 11.43 | 65 - 40 | PET-CR | 5.3 - 2.6 |
| T5 | SD | 6.8 | Yes | Yes | 5.9 | Yes | - | Yes | Yes (14) | 50.6 | 18.90 | 66 - 40 |  | - 4.3 |
| T6 | - | - | - | - | - | - | - | - | - | - | - | - |  | 1.8 - |
| T7 | PD | 1.1 | Yes | Yes | 1.1 | No | - | stable | No | 7.5 | 7.47 | 36 - 33 | PET-SD | 4.5 - 3.6 |
| T8 | SD | 6.8 | Yes | Yes | 3.3 | Yes | 5-6 | Yes | Yes (8) | 16.7 | 8.60 | 52 - 55 | PET-SD | 2.3 - 2.1 |
| T9 | SD | 4.1 | Yes | Yes | 4.1 | Yes | 2-3 | Yes | No | 19.8 | 8.20 | 42 - 61 | PET-SD | 3.3 - 2.9 |
| T10 | SD | 3.0 | Yes | Yes | 3.0 | No | - | Yes | Yes (6) | 24.0 | 11.10 | 65 - 42 | PET-SD | 1.2 - 1.3 |
| T11 | SD | 3.9 | Yes | No | 3.9 | No | 3-4 | Yes | Yes (6) | 20.7 | 14.80 | 43 - 34 | PET-SD | 2.0 - 2.5 |
| T12 | MR | 2.2 | No | Yes | 0.9 | No | - | Yes | Yes (2) | 11.6 | 2.23 | 63 - 61 | PET-PR | 5.1 - 2.6 |
| Mean (SD)/% | 9.1 % PD | n.a. | 64 % LF | 82% DF | n.a. | 55 % PsP | n.a | 82 % Yes | 64% Yes | n.a. | n.a. | 52 – 47 | 0 % PET-PD | 3.1 (1.4) - 2.6 (0.9) |
| Md (unit) | n.a. | 6.8 | n.a. | n.a. | 3.2 | n.a. | n.a | n.a. | n.a. | 24.0 | 11.3 | n.a. | n.a | 2.9 – 2.6 |
| C1 | PD | 1.5 | Yes | Yes | 1.5 | No | - | Yes | No | 20.7 | 10.37 | - | - | - |
| C2 | PD | 3.6 | Yes | Yes | 3.6 | No | - | Yes | Yes (3) | 47.6 | 40.63 | - | - | - |
| C3 | PD | 1.1 | Yes | No | 1.1 | No | - | Yes | No | 14.4 | 7.67 | - | - | - |
| C4 | SD | 2.8 | Yes | No | 2.8 | Yes | 1-3 | Yes | No | 28.7 | 19.80 | - | - | - |
| C5 | PD | 1.0 | Yes | No | 1.0 | No | - | No | No | 25.5 | 11.70 | - | - | - |
| C6 | PD | 1.0 | Yes | Yes | 1.0 | No | - | Yes | Yes (3) | 47.6 | 16.27 | - | - | - |
| C7 | PD | 1.6 | Yes | Yes | 1.6 | No | - | Yes | No | 16.2 | 3.37 | - | - | - |
| C8 | PD | 1.7 | Yes | Yes | 1.7 | No | - | Yes | No | 39.2 | 14.17 | - | - | - |
| C9 | SD | 2.1 | Yes | No | 2.1 | No |  | Yes | No | 30.7 | 11.33 | - | - | - |
| C10 | PD | 1.0 | Yes | No | 1.0 | No | - | Yes | Yes (1M) | 12.0 | 1.97 | - | - | - |
| C11 | PD | 2.2 | Yes | Yes | 2.2 | No | - | Yes | No | 17.7 | 3.40 | - | - | - |
| Mean/ % | 82 % PD | n.a. | 100 % LF | 55 % DF | n.a. | 9.1 % PsP | n.a. | 91 % Yes | 27 % Yes | n.a. | n.a. | - | - | - |
| Md(unit) | n.a. | 1.6 | n.a. | n.a. | 1.6 | n.a. | n.a. | n.a. | n.a. | 25.5 | 11.5 | - | - | - |
| P-value | **0.001** | **< 0.001** | **0.027** | 0.414^*^ | **< 0.001** | **0.063** | n.a. | 0.214 | 0.198 | 0.430 | 0.915 | n.a. | n.a. | n.a. |

*Abbreviations: RANO = Response assessment neuro-oncology criteria; CR = complete response = 1; PR = partial response=2 ; MR = minimal response; SD = Stable disease = 3; PD = Progressive disease = 4; FFLP = freedom from local tumor progression, LF = local failure, PFS = progression free survival, PsP = pseudoprogression, OS = overall survival; ndGBM = newly diagnosed glioblastoma, rGBM = Recurrent Glioblastoma; QOL FACT-BR = Quality of Life Functional Assessment of Cancer Therapy for Brain cancer patients scale (0-100); f-up = follow-up; PET = Positron emission tomography; SUVmax = standardized maximum uptake values; n.a. = not applicable; P-value = two-sided test comparing trial patients with controls using the Wilcoxon-Mann-Whitney test and *Pearson’s exact chi-square test Final*

# **Supplemetary References**

1. Saleh MG, Rimbault D, Mikkelsen M, Oeltzschner G, Wang AM, Jiang D, et al. Multi-vendor standardized sequence for edited magnetic resonance spectroscopy. Neuroimage. 2019;189:425-31.

2. Edden RA, Puts NA, Harris AD, Barker PB, Evans CJ. Gannet: A batch-processing tool for the quantitative analysis of gamma-aminobutyric acid-edited MR spectroscopy spectra. J Magn Reson Imaging. 2014;40(6):1445-52.

3. Lin A, Andronesi O, Bogner W, Choi IY, Coello E, Cudalbu C, et al. Minimum Reporting Standards for in vivo Magnetic Resonance Spectroscopy (MRSinMRS): Experts' consensus recommendations. NMR Biomed. 2021;34(5):e4484.
